# Dissection of Cervical Lymph Node Metastasis With Internal Jugular-Subclavian Venous Junction Invasion Via an Approach Involving Resection of the Margin of the Medial Clavicle

**DOI:** 10.7759/cureus.16055

**Published:** 2021-06-30

**Authors:** Takayuki Imai, Yukinori Asada, Ko Matsumoto, Takahiro Goto, Kazuto Matsuura

**Affiliations:** 1 Head and Neck Surgery, Miyagi Cancer Center, Natori, JPN; 2 Diagnostic Radiology, Miyagi Cancer Center, Natori, JPN; 3 Diagnostic Radiology, Seiryo Clinic, Sendai, JPN; 4 Plastic and Reconstructive Surgery, Miyagi Cancer Center, Natori, JPN; 5 Head and Neck Surgery, National Cancer Center East, Kashiwa, JPN

**Keywords:** head and neck cancer, hypopharyngeal cancer, neck dissection, surgical procedure, clavicle

## Abstract

We report here a patient with a massive lymphatic metastasis involving the internal jugular-subclavian venous (IJ-SCV) junction that was safely resected with a new surgical procedure without significant complications. The patient, a 57-year-old man, had advanced hypopharyngeal cancer that had metastasized to the left IJ-SCV junction with a considerable invasion of the vessels, seemingly precluding a conventional surgical intervention. We, therefore, devised a new minimally invasive surgical approach involving resection of the margin of the medial clavicle, which provided an open view of the operation field. This enabled severance of both subclavicular and brachiocephalic veins and removal of the tumor. All procedures were accomplished safely and there were no postoperative circulatory disturbances, including arm edema and compartment syndrome, in the ipsilateral arm. Additionally, postoperative adjuvant chemoradiotherapy was completed uneventfully.

## Introduction

Patients with advanced head and neck cancer (HNC) often present with cervical lymph node metastasis involving the internal jugular-subclavian venous (IJ-SCV) junction. Tumors that metastasize to or invade the lower neck, including the IJ-SCV junction, are prone to distant metastasis [1-2] and have a poor prognosis [3]. Additionally, because the surgical approach to this region is generally considered difficult and there is no established procedure for managing such lesions, they are often deemed to be unresectable. Thus, patients with IJ-SCV junction tumor involvement usually receive chemoradiotherapy as recommended for unresectable locally advanced HNC.

However, lymph node metastases invading the IJ-SCV junction are generally huge and the rate of local control by chemoradiotherapy is extremely low. Poor local control can result in serious morbidities that significantly impair patients’ quality of life (QOL), such as enlargement of the mass, ulceration, bleeding, foul odors, vascular rupture, and involvement of nerves such as the phrenic, vagal, and branchial plexus.

Here, we report a case of hypopharyngeal cancer with lymph node metastasis invading the IJ-SCV junction. We successfully resected it without complications, the procedure including resection of the margin of the medial clavicle to secure a good view of the IJ-SCV junction, enabling ligation of the subclavian and brachiocephalic veins, which are proximal and distal to the IJ-SCV junction, respectively.

## Case presentation

A 57-year-old man was referred to our hospital, his chief complaint being a large, rapidly increasing mass in his left neck. He had no medical history. He had smoked one pack of cigarettes per day for 32 years and was a daily drinker of alcohol. There was no evidence of other cancers. His local practitioner suspected cervical lymph node metastasis from hypopharyngeal cancer and accordingly referred him to our department. He complained of a sore throat, neck pain, and hoarseness.

**Figure 1:**
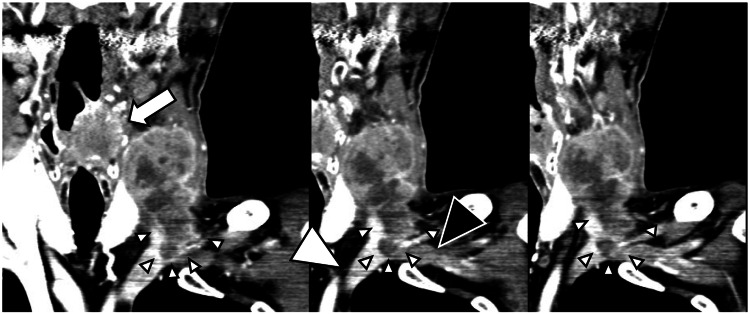
Preoperative post-contrast coronal CT images. A huge mass of metastatic lymph nodes is seen invading both the left internal jugular and subclavian veins (small arrow-heads). The left subclavian veins distal to the lesion (large black arrow-head; poor enhancement due to stagnant flow) and the left brachiocephalic vein (large white arrow-head; patent) are shown. A large primary hypopharyngeal tumor is visible (white arrow).

The primary tumor was invading a wide area from the pyriform sinus to the post cricoid and aryepiglottic fold in the left hypopharynx and was overhanging the vocal cords. Palpation revealed a large, relatively immobile mass in the left cervical level III to IV areas and the supraclavicular regions. There was no evidence of brachial plexus palsy. Diagnostic imaging revealed the primary lesion, which was accompanied by thyroid cartilage destruction, the clinical-T stage being T4a. Bilateral multiple cervical lymph node metastases were found in the right level II, III areas and the left level II, VI areas. Additionally, a huge 70-mm- × 38-mm-diameter lymph node metastasis was invading the IJ-SCV junction, this lesion being visible from the III-IV to supraclavicular areas (Figure 1). Positron emission tomography with computed tomography (PET-CT) revealed no evidence of distant metastasis.

**Figure 2:**
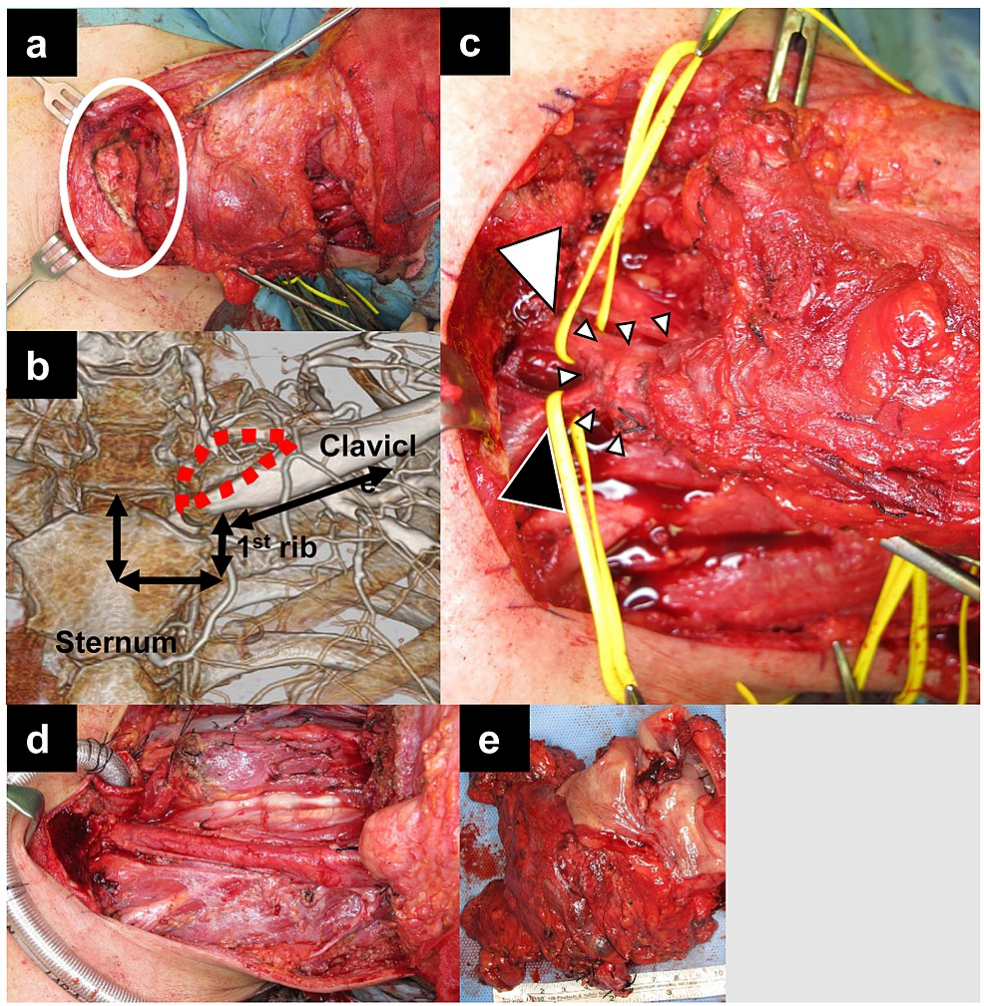
Approach for resecting tumor in the IJ-SCV junction by resecting the margin of the medial clavicle. (a) Intraoperative photograph showing resection of the margin of the medial clavicle. (b) Postoperative CT image showing clavicular margin resection approach and trans-manubrial approach. Dashed red line indicates the resected margin of the medial clavicle. Black lines indicate the resection lines in the trans-manubrial approach. Part of the sternum, first rib (at the attachment to the sternum), and soft tissue (between the first ribs and clavicle) are resected along these lines and the clavicle elevated. (c) Intraoperative photograph showing good surgical view around the IJ-SCV junction. White arrow-head indicates left brachiocephalic vein and black arrow-head indicates left subclavian vein. Small arrow-head clusters indicate the area of tumor invasion at the IJ-SCV junction. Vascular tape was applied to the brachiocephalic and subclavian veins for ligation and resection. (d) Intraoperative photograph showing neck findings after resection of tumor. (e) Photograph of the surgical specimen. IJ-SCV, internal jugular-subclavian venous

We performed total pharyngo-laryngo-cervical-esophagectomy, left hemi-thyroidectomy, bilateral neck dissection, and free jejunal transfer reconstruction. The surgical procedure for his left neck dissection is described below. The sternocleidomastoid muscle was resected, whereas the trapezius branch of the accessory nerve, hypoglossal nerve, phrenic nerve, vagal nerve, and brachial plexus nerves was preserved. The distal side of the internal jugular vein was double-ligated at the level of the posterior belly of the digastric muscle. Dissection was then advanced caudally to the supraclavicular region. The sternocleidomastoid and strap muscles, which are attached to the sternoclavicular region, were resected just above their attachments to the sternum and clavicle. The cranial half of the medial third of the clavicle was then resected using a surgical saw to enable an approach to the IJ-SCV junction (Figure 2a,b). The sternoclavicular joint was largely preserved, with only a small part of the ligament being removed. Resection of the margin of the medial clavicle dramatically improved the visibility of the IJ-SCV angle. With such an open operation field, the left subclavian and the left brachiocephalic veins were identified and double-ligated, then resected (Figure 2c). The thoracic duct was ligated and resected, the vagal and phrenic nerves being preserved. Because the lymph node metastasis was directly invading the thyroid gland, the left lobe of the thyroid gland was resected together with the level VI nodes. Dissection was carried medially toward the larynx and hypopharynx, thus completing the left neck dissection. Right conservative neck dissection and total pharyngo-laryngo-cervical-esophagectomy were subsequently performed, completing total resection of the tumor (Figure 2d,e). The total time for resection was 6 h 50 min and blood loss 224 mL and the overall total operation time 10 h 25 min with a total blood loss of 232 mL.

**Figure 3:**
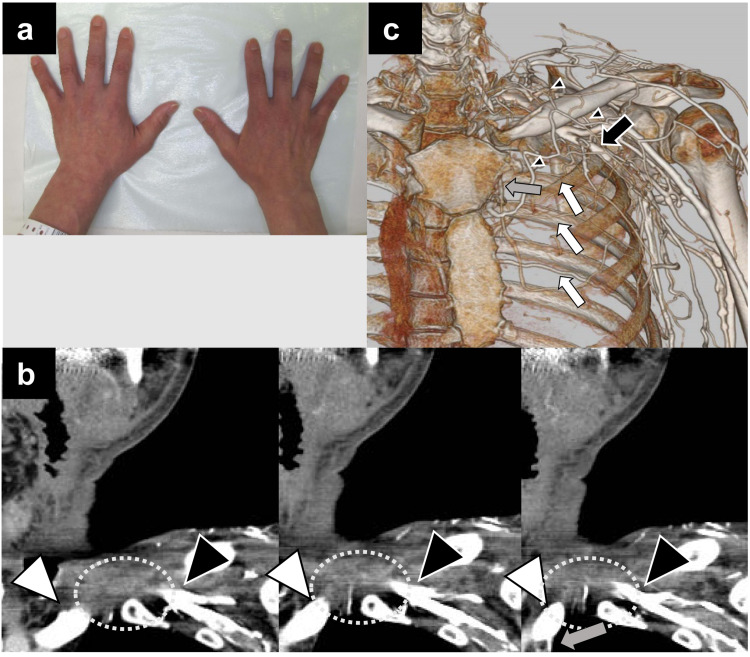
Hemodynamics after resection of brachiocephalic and subclavian veins. (a) Postoperative photograph of the hands showing there is no edema. (b) Contrast-enhanced coronal CT image showing disruption of the proximal subclavian vein (black arrow-heads) and distal brachiocephalic vein (white arrow-heads). Gray arrow indicates the inflow from the internal thoracic vein. Dashed circles indicate the tumor resection area. (c) 3-D volume rendered image with contrast medium. White arrows indicate the intercostal vein and gray arrow indicates the internal thoracic vein. Note the small blood vessels branching from the axillary vein (black arrow) to the dorsal, lower neck, and precordial regions (small black arrow-heads).

The postoperative course was uneventful without any complications. No edema was detected in his left upper extremity immediately after the surgery. The appearance of his hands two weeks postoperatively is shown in Figure 3a. Three weeks after surgery, contrast-enhanced CT was performed (Figure 3b) to check the wound site and evaluate the collateral circulation. Iodinated contrast medium was administered at a concentration of 150 mg/mL iodine and a rate of 2 mL/s via the left cubital vein, images being taken 20, 40, and 60 s after the start of injection. The left subclavian vein was found to be blocked distally. The dorsal, lower cervical and precordial regional veins were detected branching off the axillary vein, these being thought to return to the superior vena cava via the intercostal, azygos, and internal thoracic veins, respectively. Three-dimensional reconstructed CT images are shown in Figure 3c. He was discharged on the 23rd postoperative day.

Postoperative chemoradiotherapy with cisplatin and a total irradiation dose of 66 Gy was administered. No adverse effects of chemoradiotherapy, such as bone or soft tissue infection or necrosis, occurred around the site of partial resection of the clavicle. However, lumbar and lung metastases subsequently appeared. Further chemotherapy was administered, but this gradually became ineffective, and he died of disease two years and 10 months after the operation. The neck, including the left supraclavicular area, and the local area remained recurrence-free. No complications, such as drop shoulder and decreased range of motion of the shoulder joint, were detected.

## Discussion

When lymph node metastases from HNC invade the subclavian and brachiocephalic veins at the IJ-SCV junction, they are generally considered unresectable. However, there are currently no precise criteria for deeming such metastases inoperable. Further, to the best of our knowledge, no published studies have examined a large number of patients who have undergone surgery that include subclavian or brachiocephalic vein resection. Local tumor control is very important in patients with HNC because failure to achieve this results in significant impairment of patients’ QOL.

It has been reported that sternotomy is a useful approach to the upper mediastinum [4]. However, despite its relatively high degree of invasiveness, this approach rarely enables good visualization of the IJ-SCV angle, which lies laterally in the superior mediastinum. In the present case, we only needed a limited field of view around the outside of the superior mediastinal area. We considered that a trans-clavicular approach, which was originally developed for resection of pulmonary apical tumors, might provide a better view of the IJ-SCV junction. However, this approach can cause a significant decrease in QOL as a result of the deformity related to resection of the clavicle and significant impairment of shoulder function [5].

A trans-manubrial approach to apical chest tumors has also been reported [6]. Details of this procedure are described below and Figure 2b can be used as a reference. After making a longitudinal incision through the median sternum to the level of the first rib, a transverse incision is made parallel to the first rib. A longitudinal incision is then made in the first rib at the sternal attachment point, followed by a transverse incision in the tissue between the first ribs and the clavicle. This approach secures a good surgical view around the apical chest area, including the IJ-SCV junction, by elevating the clavicle while preserving the sternoclavicular joint. A few reports of using this technique to resect HNC with IJ-SCV junction invasion have been published [7-8]. In the trans-manubrial approach, the surrounding muscles, including the sternocleidomastoid muscle are also preserved. In the present case, resection of these muscles was essential because of the tumor invasion.

In contrast, the approach presented here is simpler in that only the margin of the medial clavicle is removed. Because there is an extra-nodal extension in most cases such as the current one, early postoperative chemoradiotherapy may be necessary. In the trans-manubrial approach (Figure 2b), some exudate would be expected to accumulate in the space between the reconnected bones. This increases the risk of osteonecrosis and infection related to intensive chemoradiotherapy, with total doses as high as 60 Gy or more being delivered to this area. Because osteotomy and reconnection of bones are not necessary for our approach, we consider that the risk of osteonecrosis and infection would be lower than with a trans-manubrial approach. Another advantage of our approach is that it requires no special expertise and there is no need for osteotomy of the sternum. Thus, head and neck surgeons can easily perform the procedure without assistance.

A disadvantage of this procedure is that, while it provides an open view around the IJ-SCV area, it does not provide a broad view of the operative field. Thus, it may be contraindicated when the subclavian or brachiocephalic vein needs to be followed peripherally or centrally, respectively. Of note, if the visual field proves inadequate with our approach, a trans-manubrial approach can be implemented intraoperatively. Although this procedure is simple, it has not been well reported to date. We consider it a worthwhile approach in carefully selected patients.

It is necessary to bear in mind that fracture of the remnant clavicle is a possible late complication of this procedure. A clavicle fracture is a very rare complication of neck dissection, reportedly occurring after 0.4%-0.5% of these procedures [9]. Multiple factors are considered to contribute to the development of these fractures, including extensive resection of the muscles around the clavicle during radical neck dissection, the consequent disturbance of blood flow in this area, and further deterioration in blood flow resulting from adjuvant radiotherapy and chemoradiotherapy [10]. It is impossible to estimate the incidence of fractures of the remnant clavicle after the operative procedure reported here. In contrast, the incidence of mandibular fractures associated with marginal mandibulectomy reportedly ranges from 9.1% to 13.5% [11-12]. Although the two operative procedures are not precisely equivalent, the incidence of fracture of the remnant clavicle after the present procedure will likely be similar. The indications for this procedure require careful consideration in older patients with preoperative clavicular fragility, patients taking steroid hormones regularly, and patients who have previously undergone radiation therapy to the head and neck. Precautionary measures include avoiding bruising in the area around the resected part of the clavicle. The blood flow around the clavicle must also be considered when selecting the site of skin incision in this operation. Wound dehiscence and superficial infections could result in infection of the remnant clavicle, leading to complications such as its fracture. Apron incision is often preferred for pharyngo-laryngo-cervical-esophagectomy with neck dissection [13]. In the present case, we selected an incision line similar to that for an apron incision. We consider it essential to avoid making an incision directly above the clavicle or creating a sharp angle between the incision lines and to raise the flap at an appropriate layer.

Subclavian vein ligation is rarely performed in HNC surgery; therefore, head and neck surgeons may be anxious about the resultant impairment of blood flow from the upper extremities which may lead to compartment syndrome and edema. However, subclavian vein ligation is reportedly well tolerated in almost all patients [14]. In the present case, there was no evidence of edema or compartment syndrome immediately after the operation. A subsequent contrast-enhanced CT with contrast injected into the cubital vein demonstrated a collateral circulation in which branches of the axillary vein returned to the superior vena cava via the intercostal vein to the azygos vein or via the internal thoracic vein.

Finally, although our patient ultimately died of distant metastasis, it is significant that local control was achieved by radical resection, including the IJ-SCV junction. Aggressive surgical resection prevented some of the symptoms that can significantly impair a patient's QOL, such as bleeding, foul odors, masses, and brachial plexus disorders.

## Conclusions

Here we report a case of resection of massive lymph node metastasis at the IJ-SCV junction that was enabled by achieving a good visual field. Local control is important in HNC treatment and this less invasive approach involving resection of the margin of the medial clavicle may be worth considering in selected cases with IJ-SCV involvement.
